# Radix Paeoniae Rubra and Radix Paeoniae Alba Attenuate CCl_4_-Induced Acute Liver Injury: An Ultra-Performance Liquid Chromatography-Mass Spectrometry (UPLC-MS) Based Metabolomic Approach for the Pharmacodynamic Study of Traditional Chinese Medicines (TCMs)

**DOI:** 10.3390/ijms131114634

**Published:** 2012-11-09

**Authors:** Rui Wang, Ai-Zhen Xiong, Zhong-Qiu Teng, Qi-Wei Yang, Yan-Hong Shi, Li Yang

**Affiliations:** 1 School of Pharmacy, Shanghai University of Traditional Chinese Medicine, Shanghai 201203, China; E-Mails: ellewangtcm@gmail.com (R.W.); semw521@sina.com (Q.-W.Y.); yhs_lucky@163.com (Y.-H.S.); 2 The Ministry of Education (MOE) Key Laboratory for Standardization of Chinese Medicines, Institute of Chinese Materia Medica, Shanghai University of Traditional Chinese Medicine, Shanghai 201203, China; E-Mail: a.z.xiong@hotmail.com; 3 The State Administration of Traditional Chinese Medicine (SATCM), Key Laboratory for New Resources and Quality Evaluation of Chinese Medicines, Institute of Chinese Materia Medica, Shanghai University of Traditional Chinese Medicine, Shanghai 201203, China; 4 School of Life Science, Beijing Institute of Technology, Beijing 100081, China; E-Mail: zhongqiuteng@163.com

**Keywords:** Radix Paeoniae Alba, Radix Paeoniae Rubra, UPLC-MS, metabolomics, liver injury

## Abstract

Metabolomics has been frequently used in pharmacodynamic studies, especially those on traditional Chinese medicine (TCM). Radix Paeoniae Alba and Radix Paeoniae Rubra are popularly used in TCM, and both have hepatoprotective effects. In this study, a CCl_4_-induced acute liver injury rat model was established and confirmed by the observed serum aminotransferase activities. The metabolomics approach was applied to study the influence of Radix Paeoniae Alba and Radix Paeoniae Rubra on the metabolic changes in rats with acute liver injury. The partial least-squares-discriminant analysis (PLS-DA) of rat serum and their ultra-performance liquid chromatography-mass spectrometry (UPLC-MS) fingerprints allowed discrimination of controlled, acute liver injury-model rats after administration of the two types of TCMs. The time-dependent PLS-DA plots showed that the changes in the metabolic patterns of the rats, which were administered with the TCMs, had stabilized within 2 h after they received the intraperitoneal CCl_4_ injection. The results indicated the protective effect of TCMs against liver injury. Several potential biomarkers were detected and identified, which included creatine, deoxycholic acid, choline, 5-methylenetetrahydrofolate, folic acid, and glycocholic acid. The physiological significance of these metabolic changes was discussed.

## 1. Introduction

Traditional Chinese medicine (TCM) is one of the oldest continuously practiced systems of herbal medicines in the world. TCM, which is a holistic approach to health that attempts to bring the body, mind, and spirit into harmony, has been applied for thousands of years [1]. TCM has gained widespread and increasing international interest for its potential utilization as a source of new drugs for Western markets. However, a major challenge is the insufficient scientific research on TCM caused by the lack of modern technological approaches, which has severely hindered the globalization and modernization of TCM [2].

Metabolomics, a key component of “systems biology” [3], has been considered as an important tool for understanding the biology of an organism and its response to environmental stimuli or genetic perturbation [2]. As a novel holistic approach to study metabolic profiles, metabolomics adopts a “top-down” strategy to show the biofunctions of organisms based on the terminal symptoms of metabolic networks and to understand the metabolic changes in a system that are caused by interventions [4,5]. Metabolomics has attracted increasing attention in various areas of drug research such as pharmacodynamic mechanisms [6] and toxicity [7]. Metabolomics has also been used in the investigation of the properties of TCMs [8]. Based on the paradigm that focuses on the emergent functions at the whole body level, which is consistent with the spirit of TCM, metabolomics has a great potential for harmonizing ancient TCM with modern medicine, thereby promoting the international development of TCM.

Radix Paeoniae Rubra (RPR) and Radix Paeoniae Alba (RPA) are TCMs that were firstly recorded in *Shen Nong’s Chinese Materia Medica*, which was written in the Eastern Han Dynasty (A.D. 25−220). The former is the dried root of either *Paeonia lactiflora* Pall. or *Paeonia veitchii* Lynch. RPR is traditionally used to reduce fever, eliminate stasis, activate blood circulation, and relieve pain directly. The latter is the decorticated and boiled dried root of *P. lactiflora* Pall. [1,9]. RPA is used to calm liver wind, relieve pain, nourish blood, regulate menstrual functions, and suppress sweating [10]. The hepatoprotective effects of these TCMs have been previously determined. A well-defined model was introduced in this study to evaluate their hepatoprotective effects. CCl_4_ is a well-known compound for the chemical induction of liver injury, which causes the production of free radicals in the liver after CCl_4_ metabolism. These free radicals include the trichloromethyl radical (·CCl_3_) and its peroxyl radical (·OOCCl_3_). These free radicals initiate a chain reaction of lipid peroxidation and cause subsequent cell damage [11–13]. A rat model for acute liver injury was induced by CCl_4_ in our previous work [14], and CCl_4_ hepatotoxicity was confirmed by serological examination. Metabolomic analyses, which are based on gas chromatography-mass spectrometry (GC-MS) and liquid chromatography-MS (LC-MS), have been performed on animal models with CCl_4_-induced liver injury [15,16]. Several endogenous compounds such as taurine-conjugated bile acids were identified and used as the biomarkers for hepatotoxicity, thereby confirming the potential of metabolomics in hepatotoxicity investigations. In this study, metabolomics was applied for the first time to study the hepatoprotective effects of RPR and RPA against CCl_4_-induced liver injury using an ultra-performance liquid chromatography-MS (UPLC-MS) approach. Thus, this study illustrates the effectiveness of these ancient TCMs using modern technology.

## 2. Results and Discussion

### 2.1. Hepatoprotective Effect of RPA and RPR against CCl_4_-Induced Injury

The extent of acute CCl_4_-induced rat liver injury was examined by measuring the serum alanine aminotransaminase (ALT) and aspartate aminotransferase (AST) activities. ALT and AST are common aminotransferases used in clinical chemistry to evaluate hepatotoxicity. In CCl_4_-induced rats, the ALT and AST activities in the serum were both significantly increased by 14-fold compared with the corresponding olive oil-treated controls (VEH). This difference indicated the progressive hepatic injury after CCl_4_ exposure. The serum AST and ALT activities in rats that were treated with RPR and RPA extracts were both significantly reduced by 50% to 70% compared with the CCl_4_-induced rats. However, these activities were still relatively higher than those of the control group (Table 1). These results indicated that TCMs had excellent hepatoprotective effects against acute liver injury induced by CCl_4_.

### 2.2. Multivariate Analysis

**Figure 1:**
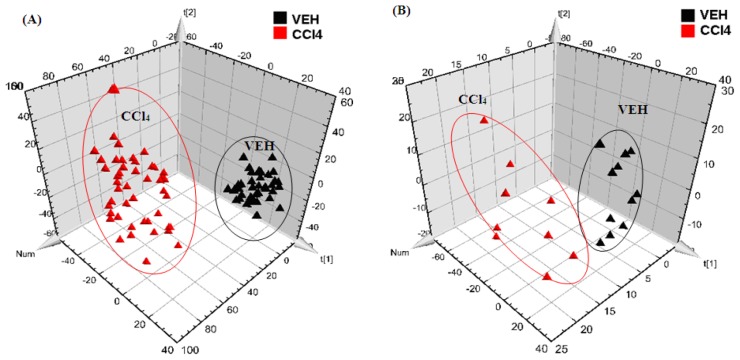
Partial least squares-discriminant analysis (PLS-DA) scores plot of the olive oil-treated controls (VEH) and the CCl_4_-induced liver injury rat model (CCl_4_) group at 24 h after the intraperitoneal injection of CCl_4_, which was generated by: (**A**) metabolomic fingerprinting analysis data and (**B**) bile acid profiling analysis data (▲ VEH group, 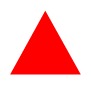 CCl_4_ group).

**Figure 2:**
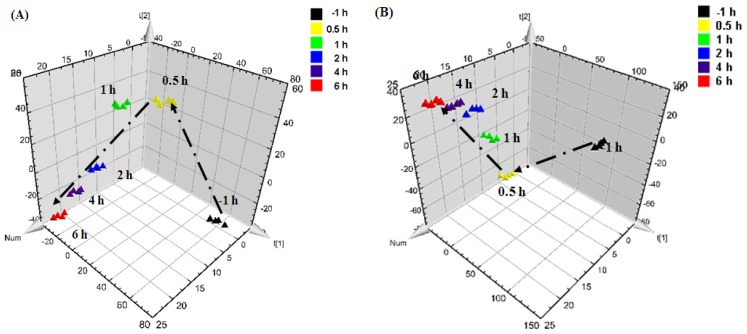

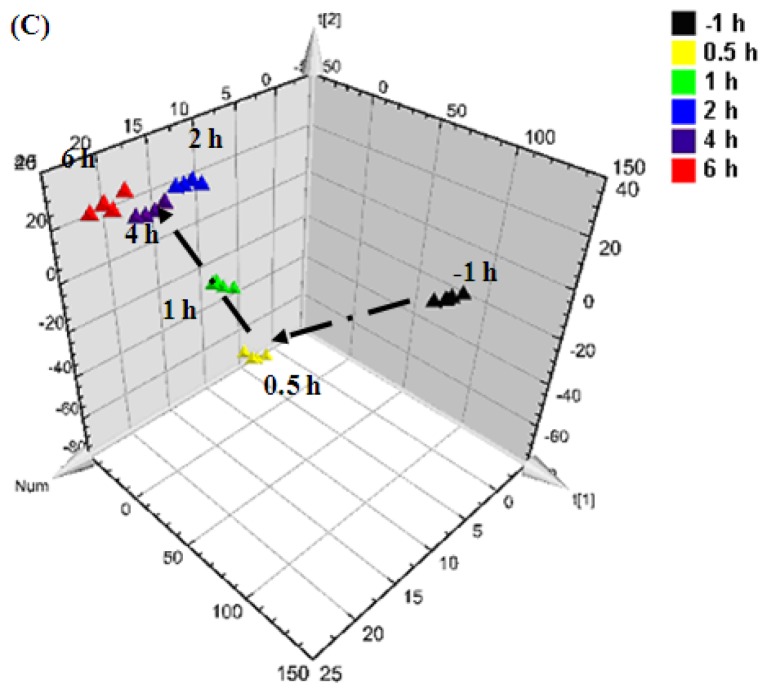
Trajectory analysis of (**A**) the CCl_4_-induced liver injury rat model group; (**B**) Radix Paeoniae Rubra (RPR)-treated group, and (**C**) Radix Paeoniae Alba (RPA)-treated group (▲ 1 h before injection, 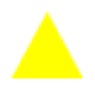 0.5 h after injection, 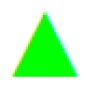 1 h after injection, 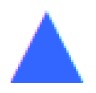 2 h after injection, 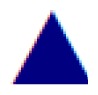 4 h after injection, and 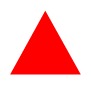 6 h after injection).

Partial least squares-discriminant analysis (PLS-DA) of the metabolomic fingerprinting data was performed to reveal the clustering information among groups. First, the CCl_4_-induced liver injury model was studied. As shown in Figure 1A, the distinct clustering of the CCl_4_ and VEH groups was seen at 24 h after the intraperitoneal CCl_4_ injection, with excellent modeling and prediction parameters (*R*^2^Y = 0.996, *Q*^2^ = 0.845). This result was consistent with the observed serum aminotransferase activities and confirmed that the endogenous compounds were greatly disrupted in the hepatotoxic group. Moreover, metabolomic trajectory analysis was also performed on the CCl_4_ group at 1 h before as well as 0.5, 1, 2, 4, and 6 h after the intraperitoneal injection of the toxin (Figure 2A). Serum samples that were collected prior to the CCl_4_ treatment were grouped in the lower right corner of the plot. With increasing time, the points gradually shifted towards the direction of decreasing *t*_2_ and increasing *t*_3_, thereby suggesting a time-dependent change in the serum’s metabolomic profile.

Dynamic PLS-DA analysis was likewise performed for the RPR- and RPA-treated groups (Figure 2B,C) from 1 h before to 6 h after the CCl_4_ treatment. The samples from both groups followed similar trends at different time points. The data points moved towards the direction of increasing *t*_2_ and *t*_3_, which was in the opposite direction compared with the trend of the CCl_4_-induced hepatotoxic model (Figure 2A). The changes in the metabolites are known to reflect the changes in the state of an organism. The trajectory analysis suggested a reversed effect on the organism when the CCl_4_ treatment was pretreated by RPR or RPA. Furthermore, the samples that were collected at 2, 4, and 6 h were clustered together with minor differences, thereby indicating that the changes in the metabolic pattern were stabilized at 2 h after the rats received the intraperitoneal CCl_4_ injection.

### 2.3. Identification of Potential Biomarkers

The serum samples of all four groups, which were collected at 24 h after CCl_4_ or controlled olive oil treatment, were analyzed by PLS-DA. The samples of the RPR- and RPA-treated groups were notably located between those of the VEH and CCl_4_ groups, which was consistent with the results of serum ALT and AST testing. The loadings plot and the variable importance in the projection (VIP) statistics of the model were further analyzed to search for potential hepatotoxic biomarkers, which contributed most to the discrimination among the groups. The variables that were situated far from the origin in the scores plot, which had a VIP value >1.0, are usually recognized as candidate biomarkers [17]. In the present study, eight compounds were selected for tandem MS (MS/MS) analysis to elucidate their structural information. The structures were temporarily identified by comparing their MS data and MS/MS fragments with those reported in the literature [15,16] and public databases, including HMDB (http://hmdb.ca), KEGG(http://www.genome.jp/kegg), and LIPID MAPS (http://www.lipidmaps.org/).

Among these compounds, four potential biomarkers had MS data, MS/MS fragments, and retention times that matched with those of tauroursodeoxycholic acid (TUDCA), glycocholic acid (GCA), cholic acid (CA), and deoxycholic acid (DCA) standard compounds. Thus, these compounds were unambiguously identified as bile acids (Table 2). The relative levels of all eight biomarkers in the two TCM-treated groups were compared with those in the VEH group using their MS intensities. The bile acid levels were most significantly altered and increased in both treatment groups, thereby indicating the importance of bile acids and suggesting the need for further investigation of these endogenous compounds.

Besides, decreased levels of choline and 5-methylenetetrahydrofolate were likewise observed in the CCl_4_ group (Table 2). Choline is a component of lecithin and is the precursor of acetylcholine compounds, which can affect a variety of metabolic processes as a methyl donor (lipid metabolism, for example). Choline deficiency is an important factor that leads to hepatotoxicity. On the other hand, 5-methylenetetrahydrofolate is the most biologically active form of vitamin B, which functions as a methyl donor in homocysteine and methionine conversion. 5-Methylenetetrahydrofolate deficiency may lead to increased cellular homocysteine, which could cause nerve injury, inflammation, and liver cell damage, or even induce cell toxicity and cancer [18]. In addition, 5-methylenetetrahydrofolate is involved in the synthesis of 5-hydroxytryptamine (5-HT, serotonin), which is a potent inducer of the early-response gene cyclooxygenase 2 (Cox-2) [19], thereby increasing the inflammatory response in rats. As revealed by the metabolomic fingerprinting analysis in the present study, the serum levels of choline and 5-methylenetetrahydrofolate in the RPR and RPA groups had the tendency to recover to normal levels, although these values were still relatively lower than those of the VEH group. The results indicated that the two TCMs could likewise achieve their hepatoprotective effect by increasing the concentrations of methyl donors to reduce intrahepatic homocysteine accumulation.

### 2.4. Bile Acid Profiling Analysis

The concentrations of each bile acid were determined according to our previously reported method [11] (Figure S2). Compared with the VEH group, the serum profiles of the bile acids were significantly changed in the CCl_4_ group (Table 3). The levels of both taurine-conjugated and free bile acids, including CA and LCA, were significantly elevated. Among these acids, CA had a six-fold increase. Several taurine-conjugated bile acids (such as TCA, TCDCA, TUDCA, THDCA, and TDCA) were elevated by at least two-fold. The TCA level, in particular, had a 20-fold increase. Moreover, GCA was elevated by 10-fold in the CCl_4_ group. LCA, which is the most hepatotoxic free bile acid, was among the most significantly elevated free bile acids in the CCl_4_ group. However, the LCA level in both the RPA and RPR groups almost recovered to the level of the VEH group. Likewise, the CA level in the RPA groups decreased, similarly to that of the VEH group. The taurine-conjugated bile acids, which were greatly elevated in the CCl_4_ group, also showed trends of recovery (Table 3).

**Figure 3:**
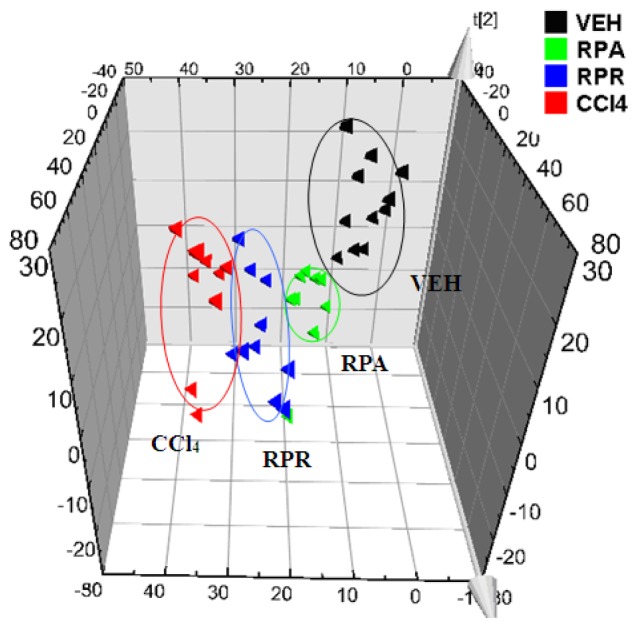
PLS-DA scores plot of rat serum samples from all the four groups, which were collected at 24 h after the intraperitoneal injection of CCl_4_, as generated from the bile acid profiling analysis data (▲ VEH group, 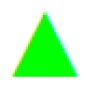 RPA group, 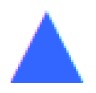 RPR group, 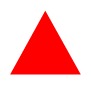 CCl_4_ group).

**Figure S3:**
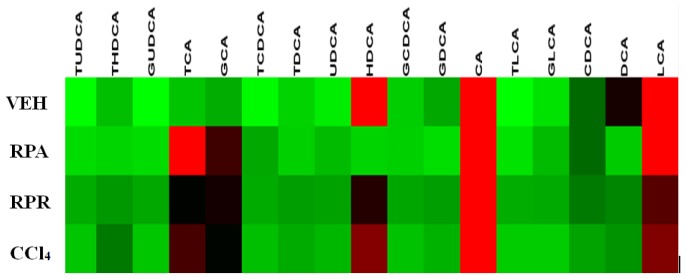
Heat map of bile acids in serum from rats of the CCl_4_, RPR, RPA, and VEH groups collected at 24 h after intraperitoneal injection of CCl_4_. (The concentrations of the bile acids are represented by colors ranging from light green to dark red. LCA, GCA, TCA, TCDCA, TDCA, and TLCA were significantly altered among groups and showed the recovery potential of the TCM-treated groups).

Multivariate statistical analysis, including PLS-DA and heat map analysis, were performed to further investigate the metabolic profile of these bile acids in order to discriminate the four treatment groups. The PLS-DA models were generated based on the serum level of bile acids between the VEH and CCl_4_ groups (Figure 1B) as well as among the four experimental groups at 24 h after CCl_4_ treatment (Figure 3). Similar to the PLS-DA analysis of the serum metabolomic fingerprinting data, all four groups were clearly separated, with the two TCM-treated groups between the VEH and CCl_4_ groups. These results indicated that RPA and RPR treatment could both change the bile acid profile towards that of the VEH group. Combined with the results of tests for serum aminotransferase activities, which proved the hepatoprotective effects of RPA and RPR, the attenuation of bile acids had a positive correlation with the detoxifying effect of TCMs against CCl_4_-induced liver injury. Furthermore, the heat map (Figure S3) clearly showed that levels of LCA and GCA, as well as the taurine-conjugated bile acids, were significantly altered among the groups, thereby implying the recovery potential of the TCM groups towards the bile acid levels of the VEH group.

The metabolomic results revealed significantly elevated concentrations of bile acids after CCl_4_ exposure, especially for the free and taurine-conjugated bile acids. As the major products of cholesterol catabolism, bile acids are essential for the solubilization and transport of dietary lipids [20]. In liver disease cases, the whole bile acid profiles change. The intrahepatic accumulation of bile acids, especially hydrophobic bile acids like LCA, could result in severe liver injury [21]. Thus, bile acids are considered to be very important diagnostics of liver diseases. In the present work, the concentrations of several bile acids were significantly increased in the CCl_4_ group but were attenuated towards normal levels in the RPR and RPA groups. These acids included LCA, CA, and several taurine-conjugated bile acids. Both RPR and RPA were proved to be hepatoprotective by testing the serum ALT and AST activities. Correlation analysis between each bile acid level and the observed ALT or AST activity was performed (Table S1). Significant positive correlations were found between CA and several taurine-conjugated bile acids (including TCA, TDCA, TCDCA, TUDCA, and THDCA) with ALT and AST, thereby indicating the importance of bile acids in the hepatoprotectivity of the two TCMs. Especially, according to not only the bile acids levels but the result of serum ALT and AST activities, the RPA group was located much closer to the VEH group in the PLS-DA score plot and with lower ALT and AST activities. These findings indicated that RPA extract showed a slightly stronger hepato-protective effect than that of RPR extract, which is consistent with the traditional usage of RPR to calm the liver. Likewise, the results showed the potential use of bile acids as new biomarkers in both the evaluation and prediction of liver injury.

## 3. Experimental Section

### 3.1. Chemicals and Materials

Acetonitrile, methanol, glacial acetic acid, and ammonium acetate (chromatographic grade) were purchased from Fisher-Scientific (Fair Lawn, NJ, USA). Carbon tetrachloride (CCl_4_), ethanol, and olive oil (analytical grade) were purchased from the Sinopharm Chemical Reagent Co., Ltd. (Shanghai, China). Distilled water, which was prepared using a Milli-Q water purification system (Bedford, OH, USA), was used throughout the study.

RPR was purchased from the Yang He Tang Pharmaceutical Co., Ltd. (Shanghai, China; No. bs20100301). RPA was obtained from the Sichuan Chinese Herbal Medicine Co., Ltd. (Chengdu, China; No. cs20100101). Both TCMs were authenticated by Dr. Wu Lihong of the Shanghai University of Traditional Chinese Medicine (SHUTCM; Shanghai, China). Both TCM extracts were prepared using the following procedure: 3.0 kg of materials were dissolved in eight times the volume of 80% alcohol, and then heated at reflux for 2 h for a total of three times. The extracts were pooled and vacuum dried at 60 °C. The extraction yields were 14.8% and 38.4% for RPR and RPA, respectively.

### 3.2. Animal Experiments

Male Sprague-Dawley rats (age, six weeks; bodyweight, 200 g ± 20 g) were obtained from the Laboratory Animal Center of the SHUTCM. The animal welfare practices and animal experimental protocols were strictly consistent with the Guide for the Care and Use of Laboratory Animals [22] and the related ethics regulations of SHUTCM. The rats were maintained in a controlled environment with a temperature of 22 ± 2 °C and a relative humidity of 55 ± 5% under a 12 h light/dark cycle. The animals were acclimatized for one week before the experiments. The rats were randomly divided into four groups and fasted for 16 h before CCl_4_ treatment.

Group 1, 2, and 3 (10 rats per group) received an intraperitoneal injection of 1 mL/kg body weight CCl_4_ (10% solution in arachis oil) after an oral dose of 4.6 g/kg RPR extract, 1.8 g/kg RPA extract, equivalent to dry crude drug 12 g/kg, or the same volume of distilled water, respectively, once a day for six consecutive days. Group 4, the control group, was given intraperitoneal injections of 1 mL/kg body weight olive oil after the oral administration of distilled water once a day for six consecutive days.

### 3.3. Sample Collection and Pretreatment

Blood samples were collected at 1 h before the intraperitoneal injection of CCl_4_ as well as 0.5, 1, 2, 4, 6, and 24 h after the injection. All blood samples were allowed to coagulate for 2 h at room temperature before they were centrifuged at 4000 rpm for 10 min at 4 °C. The serum samples were stored at −80 °C prior to any further sample preparation and analysis.

To precipitate the proteins, 100 μL of rat serum was mixed with 400 μL of acetonitrile-methanol (3:1) mixed solution, followed by 5 min of vortex mixing. After centrifugation at 12,000 rpm for 15 min, the protein extracts were then kept at 4 °C. A 5 μL aliquot of the supernatant was then injected into the UPLC-MS system for analysis.

### 3.4. Assay of Serum Aminotransferase Activities

The ALT and AST activities of serum samples were enzymatically determined from the rate of NADH oxidation using kits obtained from the Stanbio Laboratory (Boerne, TX, USA).

### 3.5. Metabolomic Fingerprinting Analysis

A Waters ACQUITY UPLC system coupled with a ZQ2000 single quadrupole mass spectrometer (Waters Corp., Milford, MA, USA) was used to obtain the entire fingerprint of each serum sample (Figure S1). A Waters Premier triple-quadrupole tandem mass spectrometer was used for the fragmentation of the targeted endogenous metabolites. Chromatographic separation was performed at 45 °C on a Waters HSS T3 column (1.7 μm particle size, 2.1 mm × 100 mm internal diameter) with a gradient elution of solvent A (acetonitrile) and solvent B (0.1% formic acid containing 5 mM ammonium acetate) as follows: 98% B (0.0–1.0 min), 98% to 50% B (1.0–3.0 min), 50% to 40% B (3.0–12.0 min), 40% to 10% B (12.0–16.0 min), 10% B (16.0–17.0 min). The mobile phase was kept at 98% B for another 3 min before the next injection. The injection volume was 5 μL, and the flow rate was set at 0.3 mL/min. All mobile phase solvents were filtered with a Whatman 0.22 μm nylon filter.

The mass spectrometer was operated in the electrospray ionization (ESI) negative-ion mode with full scan function. The capillary voltage was optimized to 5.0 kV, whereas the cone voltage was 38 V. The source and desolvation temperatures were set at 120 and 300 °C, respectively. The desolvation and cone gas flow rates were set at 700 and 50 L/h, respectively. For the MS/MS analysis of candidate biomarkers, the collision energy was set from 20 eV to 35 eV. The UPLC-MS and MS/MS data were acquired and processed using the MassLynx 4.1 software (Version SCN 704; Waters Corp.: Milford, MA, USA).

### 3.6. Metabolomic Profiling Analysis of Bile Acids

The samples were prepared by diluting 200 μL of serum with 600 μL of methanol, following our previously reported method [11]. The prepared samples were applied to a Waters ACQUITY UPLC system coupled with a ZQ2000 quadrupole mass spectrometer at the ESI negative mode for the metabolomic profiling analysis of the bile acids.

### 3.7. Data Processing and Statistical Analysis

The metabolomic fingerprinting data of the serum samples were extracted, aligned, and normalized using the MarkerLynx software (Waters Corp.) to generate a three-dimensional matrix containing the arbitrarily assigned peal index (retention time_*m*/*z* pairs), sample name (observations), and ion intensity information (variables). The following typical parameters for a single quadrupole mass spectrum were set: the retention time (*t*_R_) ranged from 0.5 min to 17.0 min, the mass ranged (*m*/*z*) from 100 to 1000, with an extracted ion chromatogram window (the mass tolerance) of 0.05 Da. The width of an average peak at 5% height and the peak-to-peak baseline noise were automatically calculated. The minimum intensity was set to 10% of the base noise. The mass and retention-time windows for marker collection were set at 0.05 Da and 0.2 min, respectively. The elimination level was set at 10.00. All isotopic peaks were excluded from the analysis.

The data sets were then analyzed by multivariate statistical analysis using the SIMCA-P^+^ version 12.0.1 software package (Umetrics AB, Umea, Sweden). PLS-DA was performed to show the natural separation among the four groups by the visual inspection of score plots. The differences between groups were explored by incorporating the VIP statistics to extract novel potential biomarker ions in the PLS-DA model.

The serum levels of bile acids were applied to the HCE3.5 software to obtain the heat map of all groups. Correlation analysis between the serum aminotransferase activity and the level of each bile acid was performed using SPSS (version 12.0; SPSS Inc.: Chicago, IL, USA).

## 4. Conclusions

A metabolomic method based on UPLC-MS and multivariate statistical techniques was developed. Together with the serum activities of aminotransaminases, this method was used to evaluate the hepatoprotective effect of RPR and RPA on CCl_4_-induced liver injury. In this study, the two TCMs were found to reduce the serum levels of several bile acids, including CA, DCA, GCA, and TUDCA, while they increased the serum levels of choline and 5-methylenetetrahydrofolate. Thus, the TCMs could modulate the homeostasis of bile acids and homocysteine in hepatocytes, thereby achieving the hepatoprotective effect. This study demonstrated that metabolomics is a powerful tool in evaluating the pharmacological effects of traditional drugs, which could lead to the discovery of underlying mechanisms of TCMs. Thus, metabolomics could promote the modernization and globalization of ancient medicines.

## Figures and Tables

**Table 1:** Serum activities of alanine aminotransaminase (ALT) and aspartate aminotransferase (AST).

Group No.	Dosage	ALT (IU/L)	AST (IU/L)
VEH	distilled water for 6 days and olive oil (1 mL/kg)	85.0 ± 4.9	195.1 ± 62.3
CCl_4_	distilled water for 6 days and CCl_4_ (1 mL/kg)	1200.0 ± 386.2 ^△△^	2790.1 ± 936.9 ^△△^
RPR	RPR (12 g/kg) for 6 days and CCl_4_ (1 mL/kg)	420.4 ± 93.1 ^▲▲^	1026.8 ± 182.5 ^▲▲^
RPA	RPA (12 g/kg) for 6 days and CCl_4_ (1 mL/kg)	316.4 ± 89.2 ^▲▲^	734.3 ± 206.1 ^▲▲^

△ significance compared with the VEH group: ^△^*p* < 0.05, ^△△^*p* < 0.01;

▲ significance compared with the CCl_4_ group: ^▲^*p* < 0.05, ^▲▲^*p* < 0.01;

VEH, olive oil-treated controls; CCl_4_, CCl_4_-induced liver injury rat model; RPR, Radix Paeoniae Rubra-treated rats; RPA, Radix Paeoniae Alba- treated rats.

**Table 2:** Identification of potential biomarkers.

*m*/*z* ([M-H]^−^)	*t*_R_ (min)	Compounds	Change in the RPR group	Change in the RPA group
269.5	4.63	Inosine	↑	↑
318.6	5.44	Phytosphingosine	↓	—
498.6	3.93	Tauroursodeoxycholic acid *	↑	↑
464.6	4.38	Glycocholic acid *	↑	↑
407.5	5.48	Cholic acid *	↑	↑
391.6	5.72	Deoxycholic acid *	↑	↑
228.5	4.11	5-Methyltetrahydrofolate	↓	↓
103.4	1.18	Choline	↓	↓

↑ content increased; ↓ content decreased; — content unchanged;

* Confirmed with standard compounds.

**Table 3:** Serum levels of bile acids (ng/mL, Mean ± SD).

Bile acids	VEH group	CCl_4_ group	RPR group	RPA group
CA	72.9 ± 60.9	474.4 ± 299.3 ***	621.7 ± 355.5 ***	141.8 ± 183.8
DCA	20.1 ± 10.5	15.6 ± 10.9	13.8 ± 9.5	2.2 ± 4.0 ***
CDCA	10.4 ± 11.0	11.2 ± 9.6	17.9 ± 10.6	11.1 ± 20.5
UDCA	1.3 ± 2.7	5.8 ± 8.7	3.8 ± 3.8	3.7 ± 6.0
HDCA	86.2 ± 51.8	112.3 ± 82.5	80.9 ± 33.9	1.3 ± 2.7 *
LCA	73.4 ± 25.9	111.2 ± 27.2 **	108.5 ± 34.0	81.4 ± 20.1
GCA	5.7 ± 7.4	54.1 ± 52.9 **	72.7 ± 40.1 ***	30.0 ± 22.0 **
GDCA	6.0 ±4.4	7.1 ± 8.9	5.4 ± 6.0	0.5 ± 1.4 *
GCDCA	3.4 ±5.1	2.8 ± 5.2	2.3 ± 3.4	1.8 ± 3.3
GUDCA	-	1.7 ± 2.8	1.6 ± 2.3 *	0.6 ± 1.1
GLCA	2.0 ± 4.6	0.2 ± 0.3	1.0 ± 1.6	3.7 ± 8.2
TCA	4.1 ± 5.2	85.9 ± 56.4 ***	60.0 ± 38.3 ***	59.3 ± 52.7 **
TDCA	0.3 ± 0.7	3.9 ± 4.6 *	0.8 ± 1.7	5.4 ± 5.5 **
TCDCA	3.1 ± 2.6	9.1 ± 7.5 *	5.2 ± 4.3	1.8 ± 3.3
TUDCA	0.2 ± 0.5	1.9 ± 2.0 *	0.3 ± 0.4	0.6 ± 0.9
THDCA	4.5 ± 8.1	22.6 ± 20.5 *	7.5 ± 5.6	1.2 ± 2.1

CA, cholic acid; DCA, deoxycholic acid; CDCA, chenodeoxycholic acid; UDCA, ursodeoxycholic acid; HDCA, hyodeoxycholic acid; LCA, lithocholic acid; GCA, glycocholic acid; GDCA, glycodeoxycholic acid; GCDCA, glycochenodeoxycholic acid; GUDCA, glycoursodeoxycholic acid; TCA, taurocholic acid; TDCA, taurodeoxycholic acid; TCDCA, taurochenodeoxycholic acid; TUDCA, tauroursodeoxycholic acid; THDCA, taurohyodeoxycholic acid.

* Compared with the VEH group,

* *p* < 0.05,

** *p* < 0.01, and

*** *p* < 0.001;

-: undetected.

**Figure A1:**
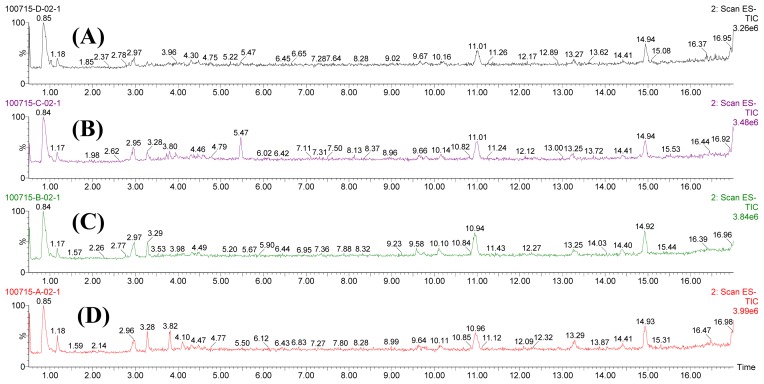
UPLC-MS total ion chromatogram of the serum samples from (**A**) the CCl_4_-induced acute liver injury rat models (CCl_4_); (**B**) Radix Paeoniae Rubra (RPR)-treated group; (**C**) Radix Paeoniae Alba (RPA)-treated group, and (**D**) the control (VEH) group after 24 h of CCl_4_ or olive oil injection.

**Figure A2:**
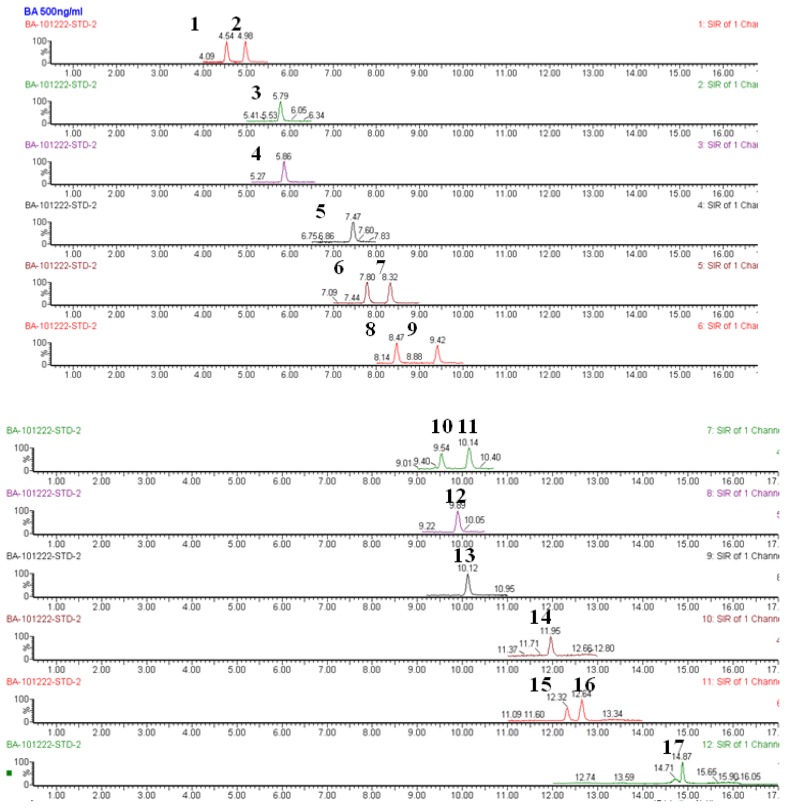
UPLC-MS-selected ion recording chromatogram of the blank serum spiked with a mixture of 500 ng/mL bile acid standards (1, TUDCA; 2, THDCA; 3, GUDCA; 4, TCA; 5, GCA; 6, TCDCA; 7, TDCA; 8, UDCA; 9, HDCA; 10, GCDCA; 11, GDCA; 12, CA; 13, TLCA; 14, GLCA; 15, CDCA; 16, DCA; 17, LCA).

## Appendix

**Table A1:** Correlation analysis between the serum levels of the bile acids and the activity of the aminotransferases, alanine aminotransaminase (ALT), and aspartate aminotransferase (AST).

Bile acid	ALT		AST	

	*r*	*p*	*r*	*p*
CA	0.385 *	0.029	0.395 *	0.025
DCA	0.095	0.605	0.093	0.614
CDCA	0.032	0.862	−0.042	0.820
UDCA	0.408 *	0.021	0.414 *	0.019
HDCA	0.408 *	0.020	0.393 *	0.026
LCA	0.271	0.134	0.255	0.160
GCA	0.300	0.095	0.333	0.062
GDCA	0.145	0.428	0.108	0.558
GCDCA	0.129	0.480	0.149	0.416
GUDCA	0.218	0.230	0.242	0.182
GLCA	−0.095	0.604	−0.099	0.591
TCA	0.542 **	0.001	0.578 **	0.001
TDCA	0.393 *	0.026	0.419 *	0.017
TCDCA	0.456 **	0.009	0.514 **	0.003
TUDCA	0.654 **	0.000	0.643 **	0.000
THDCA	0.588 **	0.000	0.611 **	0.000
TLCA	−0.156	0.395	−0.169	0.356

CA, cholic acid; DCA, deoxycholic acid; CDCA, chenodeoxycholic acid; UDCA, ursodeoxycholic acid; HDCA, hyodeoxycholic acid; LCA, lithocholic acid; GCA, glycocholic acid; GDCA, glycodeoxycholic acid; GCDCA, glycochenodeoxycholic acid; GUDCA, glycoursodeoxycholic acid; TCA, taurocholic acid; TDCA, taurodeoxycholic acid; TCDCA, taurochenodeoxycholic acid; TUDCA, tauroursodeoxycholic acid; THDCA, taurohyodeoxycholic acid; and TCLA, taurolithocholic acid. *r*, correlation factor; *p*, significance of correlation;

* significance of correlation,

* *p* < 0.05,

** *p* < 0.01.
